# Species’ traits modulate rapid changes in flight time in high-Arctic muscid flies under climate change

**DOI:** 10.1098/rspb.2025.0970

**Published:** 2025-07-09

**Authors:** Hannah Sørine Gerlich, Sarah Loboda, Gavin L. Simpson, Jade Savage, Niels Martin Schmidt, Martin Holmstrup, Toke Thomas Høye

**Affiliations:** ^1^ Department of Ecoscience, Aarhus University, C.F. Møllers Allé 4-8, Aarhus C DK-8000, Denmark; ^2^ Fisheries and Oceans Canada, Maurice Lamontagne Institute, 850 Rte de la Mer, Mont-Joli, Quebec G5H 3Z4, Canada; ^3^ Department of Animal and Veterinary Sciences, and iClimate, Aarhus University, Blichers Allé 20, Tjele DK-8830, Denmark; ^4^ Department of Biology and Biochemistry, Bishop's University, 2600 College Street, Sherbrooke, Quebec J1M 1Z7, Canada; ^5^ Department of Ecoscience, Aarhus University, Frederiksborgvej 399, Roskilde DK-4000, Denmark; ^6^ Arctic Research Centre, Aarhus University, Ole Worms Allé 1, Aarhus DK-8000, Denmark

**Keywords:** Arctic, insects, climate change, long-term monitoring, phenology, species traits

## Abstract

Insects are experiencing notable phenological shifts owing to climate change, with substantial interspecific variability. However, our understanding is limited by a shortage of long-term studies, beyond Lepidoptera. This study presents a hierarchical modelling framework to analyse the phenological distribution of 11 muscid fly species across three vegetation types over 18 years (1996–2014) in Zackenberg, Northeast Greenland. We examined species-specific changes in phenology and assessed ecological traits for explaining interspecific variation. Additionally, we investigated the associations between phenological shifts and timing of snowmelt and temperature. We found consistent trends of earlier flight activity and interspecific variation in responses, with smaller species shifting their end-of-the-season activity at faster rates than larger species. Flight activity was strongly associated with the timing of snowmelt, while warming was linked to an earlier end-of-the-season activity. Late-active species exhibited more pronounced shifts in response to climate variations than early-active species. This study highlights that the species-specific climate sensitivity of high-Arctic muscid flies potentially has demographic effects if temporal overlap among interacting species changes. We advocate for prioritizing species-specific insect population studies, ideally analysed within the context of interacting species, to understand better and address disparities in responses to climate change.

## 1. Introduction

Phenology, the seasonal timing of biological events, is a key ecological indicator of climate change [1,2]. Many studies have documented earlier spring and summer phenological events [3,4] and delayed fall events [5,6] in response to warming. The Arctic is experiencing exceptionally rapid environmental changes, with increasing temperatures and shifting snow regimes [7–9]. Arctic insects, spanning diverse taxonomic groups, display high phenological sensitivity to these changing environmental conditions [10,11].

Climate-driven phenological shifts may expose organisms to novel abiotic conditions [12,13] and alter the synchrony among interacting species if the rate of phenological change varies among species [14,15]. Phenological asynchrony, such as the timing of resource availability relative to consumer activity, can directly impact fitness and influence reproductive success or survival [2,16]. However, understanding species-specific phenological shifts is challenging owing to a lack of long-term Arctic insect records [17]. The ecosystem monitoring program at Zackenberg, Northeast Greenland, offers a rare opportunity for such studies, collecting weekly terrestrial arthropod samples since 1996 [18].

Among Arctic insects, muscid flies (Diptera: Muscidae) are particularly important pollinators and have been the focus of multiple studies on climate-driven changes in pollination networks [19–22]. Their flight period involves three key events: onset (first adult emergence), peak (maximum abundance) and end (cessation of flight)—which are all crucial for pollen transfer. These events are influenced by developmental time but they also respond to distinct environmental cues [23,24]. Beyond pollination, muscid flies play significant roles in Arctic food webs, both as prey for birds and spiders [25,26], and as predators of other invertebrates [27]. Shifts in their flight activity could disrupt synchrony with plants, prey and predators, potentially altering food web dynamics and affecting long-term population viability.

Phenological responses to climate change often correlate with functional traits [28–30]. For instance, the phenological niche—whether a species is early-active or late-active—may influence how species respond to warming [31–33], with early-active species of butterflies and bees, and early-flowering plants showing more pronounced advancements than late-active species in some environments [29,34,35]. Body size may also modulate species’ responses [3], with smaller species generally exhibiting greater phenological shifts and shorter activity periods [36,37]. Flower-visiting behaviour could further elucidate certain variations in phenological responses, as frequent flower visitors may be more constrained by the phenology of their host plants [38–40], whereas infrequent flower visitors exhibit more seasonal plasticity, allowing them to track environmental change more freely. A trait-based approach can help identify climate-sensitive species and improve our understanding of Arctic ecosystem changes [28,34].

Many high-latitude Diptera exhibit plastic responses to warming by accelerating development [41,42]. At Zackenberg, Northeast Greenland, muscid flies have advanced their flight activity with earlier snowmelt and warming [11], although the rate of these shifts has recently slowed [43]. However, shifts in flight period length vary by habitat [11,44], perhaps owing to differences in species composition [10,45,46]. While species-level analyses are crucial for understanding phenological responses [10,39], no studies have, to our knowledge, investigated interannual phenological dynamics across individual muscid species (but see Wirta *et al.* [47] for estimates of some muscid fly species’ phenological niches).

Muscid flies are among the most diverse and abundant insect families in Northern ecosystems [48–50]. In high-Arctic Greenland, most species belong to the genera *Spilogona* (Schnabl), *Drymeia* (Meigen) and *Phaonia* (Robineau-Desvoidy). Many adults visit flowers for nectar and/or pollen or to bask [27,51,52], making them important Arctic pollinators [53,54]. Notably, *Spilogona sanctipauli* (Malloch 1921), *Drymeia segnis* (Holmgren 1883) and *Phaonia bidentata* (Ringdahl, 1933) are among the most common visitors to flowers across various plant families [20,21,53,54]. In addition, species of the genus *Spilogona* also exhibit predatory or scavenging behaviours, preying on soft-bodied flies like chironomids and simulids [27]. Larvae are predominantly predatory, inhabiting semi-dry to aquatic environments, including humus-rich soil (*Drymeia* and *Phaonia* species), cushions of moss or green algae, carrion and nests of mammals and birds (*Spilogona* species) [27,55]. Adults are not strongly tied to specific vegetation types and are found in most terrestrial and freshwater environments, but some prefer the more arid habitats [44].

Here, we examined the full seasonal flight period of 11 muscid fly species across three vegetation types in Zackenberg across 18 years. Using a Bayesian hierarchical modelling framework, we accurately estimated annual onset, peak and end of phenology, capturing both nonlinear seasonal patterns and long-term trends, while maintaining the flexibility to account for variations in the shape of these patterns across different species and vegetation types [56]. Our objectives were to: (i) determine temporal trends in species-specific flight periods; (ii) assess whether these shifts correlate with temperature and snowmelt; and (iii) explore how functional traits, including phenological niche, body size and anthophilous habits (frequent versus infrequent flower visitors), predict phenological responses.

We hypothesized that species would shift flight activity earlier in response to earlier snowmelt and warming, similar to trends observed in other Arctic and Alpine insects [10,30,39,44]. However, we expected interspecific variation in these responses [10,32,45,57], partly influenced by species’ functional traits. Owing to the region’s remoteness and historical focus on more charismatic taxa (e.g. bees and butterflies), limited research on Arctic Muscid life history and ecology constrains our understanding of their ecological roles and climate responses. Therefore, we focused on traits where robust information was available and with known influences on insect phenology, including body size, phenological niche, habitat preferences and flower-visiting behaviour. We predicted that early-active species would exhibit more pronounced shifts towards earlier flight activity than late-active species, similar to other pollinators in temperate regions [31,34,58]. Conversely, we expected late-active species to delay their end-of-the-season flight activity. Smaller species were expected to exhibit stronger shifts towards earlier flight periods than larger species [3]. Habitat preference was also anticipated to shape responses, with wetland-associated species responding more strongly to climate variables, reflecting drying of wetland habitats owing to altered snow precipitation and warming [59,60]. Finally, we expected frequent flower visitors to shift less than infrequent ones, given their dependence on host plant phenology [29,40].

## 2. Material and methods

### (a) Study site and data collection

Muscid flies were collected at Zackenberg in high-Arctic Northeast Greenland (74°28' N, 20°34' W) as part of the Greenland Ecosystem Monitoring programme (see Schmidt *et al.* [18] for a detailed protocol). Between 1996 and 2014, a total of 19 807 specimens were collected across three habitat types: arid heath, mesic heath and wet fen. These specimens were identified to the species level, representing 14 species across four genera (*Spilogona*, *Drymeia*, *Limnophora* and *Phaonia*). However, only 11 species had sufficient abundances for phenology estimations. The most abundant genus was *Spilogona*, with nine species, while *D. segnis*, *S. sanctipauli* and *S. zaitzevi* (Schnabl, 1915) were the most numerous species overall (electronic supplementary material, table S1.1). Most flies were captured in the wet fen, which likely provides favourable microhabitats for many species [27]. However, *P. bidentata* and *Drymeia groenlandica* (Lundbeck, 1901) were predominantly found in the arid heath, while *D. segnis* and *S. sanctipauli* were more frequently collected in both the mesic and arid heath (electronic supplementary material, table S1.1). The wet fen was primarily dominated by mosses and grasses and has high soil moisture and early snowmelt. The mesic heath was dominated by lichens, *Cassiope tetragona*, *Dryas* spp. and *Salix arctica* and typically had snowmelt two weeks later than the fen and arid heath area. The arid heath was composed primarily of lichens, *Dryas* spp. and grasses, which had relatively low soil moisture and experienced early snowmelt. Flies were collected using yellow pitfall traps, which were deployed when the snow melted (typically late May to early June) and remained operational throughout the active season, with weekly collections until September 1st (often coinciding with freeze-up). Initially, eight traps per vegetation type were used (1997−2006), but this was reduced to four traps from 2007 onwards (four traps were also used in 1996). In 1996, samples from all traps were pooled, so we divided muscid abundances from that year by two to ensure comparability with other years, which were based on two pitfall traps per vegetation type. Samples were stored in 75% ethanol and transported to Denmark, where arthropods were sorted to family level by technicians at the Department of Ecoscience at Aarhus University. Muscid flies from two pitfall traps per vegetation type were identified to species level using morphological characters [27,55,61] and DNA barcoding. DNA barcoding [62] was used to confirm male/female associations and to corroborate identifications for a subset of specimens. Tissue samples from 61 specimens (one leg per specimen) were sent to the Centre for Biodiversity Genomics (Guelph, Ontario, Canada) for DNA extraction (https://ccdb.ca/wp/wp-content/uploads/2016/09/CCDB_DNA_Extraction.pdf), amplification of the Folmer region (656 bp) of the mitochondrial cytochrome oxidase subunit 1 gene (CO1) (https://ccdb.ca/wp/wp-content/uploads/2016/09/CCDB_Sequencing.pdf) using the C_LepFolF/C_LepFolR primers [63] and Sanger sequencing (https://ccdb.ca/wp/wp-content/uploads/2016/09/CCDB_Sequencing.pdf). DNA barcodes were successfully sequenced for 58 specimens, which were compared with reference sequences on BOLD and GenBank. A representative sequence for each species is available on Dryad [64] and were originally retrieved from BOLD Systems (Muscidae of Zackenberg (https://boldsystems.org/, dx.doi.org/10.5883/DS-MOZK (accessed March 2025)). Voucher specimens were deposited in the Bishop’s University Insect Collection (Québec, Canada). For analyses, we restricted data to years and vegetation types where at least three individuals of a given species were collected, summing individuals across all traps within a habitat and year (electronic supplementary material, figure S1.1). Temporal trend analyses were only conducted for species with sufficient data to estimate phenology in at least 5 years.

### (b) Climate variables

Throughout the study period, temperatures in soil (0, 5 and 10 cm below ground) and air (2 m above ground), alongside snow depth were recorded hourly by an automated weather station located in a mesic heath habitat and within 600 m from all plots. Timing of snowmelt was estimated as the first date when less than 10 cm of snow was consistently measured by an automatic ultrasonic snow depth sensor [65]. Years 1996, 2009 and 2013 had limited snow accumulation, resulting in the estimation of very early snow melting dates. We used soil temperature data (averaged from across 0, 5 and 10 cm depth) to estimate more reliable snow melting dates for these years, following the method in Rixen *et al.* [8]. We identified the time period towards the end of the winter when ground temperatures were stable near 0°C and subsequently started fluctuating when the snow cover disappeared (defined as the zero-curtain window). From this, we defined the date of snowmelt as when the mean daily soil temperature rose above +1°C after a period with diurnal fluctuations of less than 2 K and mean daily temperatures between −1°C and 1°C [8].

Air temperature, rather than soil temperature, was chosen to examine the relationship between temperature variations and the flight activity of flies in this study, because severe multicollinearity has previously been found between soil temperature and timing of snowmelt (average variance inflation factor>5) [11]. We initially used a sliding window approach to identify the time period during which the mean daily temperature had the strongest influence on phenological events with the *climwin* R package v. 1.2.3 [66]. However, no significant temperature window emerged. Instead, we calculated temperature as the 30-day mean preceding the species-by-plot average phenological event, adjusted by subtracting the minimum standard deviation. This approach avoids using post-event temperatures as predictors and aligns with previous studies identifying the 30-day window as the best predictor of adult insect phenology [67,68].

### (c) Analyses

#### (i) Quantifying phenology of the flight season

We used a hierarchical generalized additive model (HGAM), an empirical Bayesian approach, allowing us to simulate phenological events from the posterior distribution of the model to account for uncertainty in the model and incorporate uncertainty bands to estimates (electronic supplementary material, figure S1.2).

Weekly abundances of muscid species were modelled using HGAM with a flexible seasonal component, separating seasonal and yearly trends across species and vegetation types (when explaining the model, vegetation types are referred to as plots) [56]. The weekly abundance estimates for each year were assumed to follow a negative binomial distribution, with the global spline for seasonality indicating unimodal abundance across the growing season (electronic supplementary material, figure S1.2). A clear trend towards earlier activity over time emerged, reflecting the year component of the model. Exploratory analysis suggested substantial variation in within- and between-year trends across species and vegetation types (electronic supplementary material, figures S1.1 and S1.2). This intergroup variability could be modelled in several ways; however, we settled on the following model based on AIC values as model selection criteria (electronic supplementary material, table S1.2):


$$\begin{array}{rl} y_{i} & ∼\text{NB}(\mu_{i},\phi )\\ E(y_{i}) & =\mu_{i}\\ \log(\mu_{i}) & =f({\text{DOY}}_{i})\;+\;f({\text{Year}}_{i})+f({\text{DOY}}_{i},{\text{Year}}_{i})\;+\\ & \;f_{\text{Year}\left(i\right)}({\text{DOY}}_{i})+f_{\text{Plot}(i),\;\text{Species}(i)}({\text{DOY}}_{i},{\text{Year}}_{i})\;+\\ & \;\gamma_{\text{Plot}(i)}+\xi_{\text{Species}(i)}+\log({\text{exposure}}_{i}) \end{array}$$

where the observed abundances, *y_i_*, of muscid flies are modelled as random variables following a negative binomial distribution, with expected value *µ_i_* and dispersion parameter ϕ. The log of the expected abundance, log(𝜇_𝑖_), is modelled as a sum of smooth functions. The first three functions, as defined in Pedersen *et al.* [56], represent average or global smooths of day of year (𝑓(DOY_𝑖_)), year (𝑓(Year_𝑖_)) and their smooth interaction (𝑓(DOY_𝑖_, Year_𝑖_)). These functions capture common seasonal and between-year trends, with the interaction allowing for the seasonal trend to vary smoothly over years. To model individual years’ seasonal effects, we use a random smooth of the day of year (𝑓_Year(𝑖)_(DOY_𝑖_)), enabling greater between-year variation that can be captured by the three average smooths. To model variation in seasonal and between-year trends within species and vegetation types (plots), a tensor product smooth with four marginal smooths is used [69]. These include (i) day of year, (ii) year, (iii) random effect for vegetation type, and (iv) random effect of species (𝑓_Plot__(𝑖),__Species__(𝑖)_(DOY_𝑖_, Year_𝑖_)). Random intercepts are used to allow the abundance of muscid flies to vary among vegetation types (𝛾_Plot__(𝑖)_) and species (𝜉_Species__(𝑖)_). To account for the exposure time of the traps and convert our model into one for *daily* muscid fly abundance, an offset term in the form of log(exposure_𝑖_) is included, where exposure_𝑖_ represents the number of days during which the trap was exposed for each observed count. Each observation is indexed through subscript *i*, with Year_(𝑖)_, Plot_(𝑖)_, and Species_(𝑖)_ indicating the year, vegetation type and species to which the *i*^th^ observation belongs. Smoothing parameter estimation is carried out through REML (restricted maximum likelihood [70]) using techniques for large GAMs with covariate discretization [71–73]. Smooth functions are represented in the model using cubic spline with second derivative penalties.

We identified the peak week of each year when abundance of flies was at the maximum predicted by the model. For each of the 10 000 posterior draws, we extracted the maximum of the estimated seasonal abundance curve for each vegetation type, year and species combinations and stored the day of the year on which this maximum occurred. This yielded a posterior distribution of 10 000 values for the peak day of year for each species in each vegetation type and year. Credible intervals (0.025 and 0.975 probability quantiles) for the annual seasonal peaks were computed from these posterior distributions. Onset and end of activity were estimated by calculating the cumulative sum of daily estimated abundance for each posterior draw for each combination of species, vegetation type and year. Onset was defined as the first day of the year when the cumulative abundance equalled or exceeded 10% of the annual sum, and end as the first day of year when the cumulative sum equalled or exceeded 90% of the total individuals. This threshold-based approach reduces the influence of low-abundance tails, which are more susceptible to interannual population fluctuations and sampling error [74,75], ensuring a more robust and ecologically meaningful estimate of seasonal activity. Uncertainty around these estimates was calculated by using the 0.025th and 0.975th probability quantiles of the posterior distribution for each combination of species, vegetation type and year, identical to the approach for peak activity.

All statistical analyses were performed in R v. 4.4.1 [76]. The R package ‘*mgcv*’ v. 1.9−0 [72] was used to fit all models using HGAM and the package ‘*gratia*’ v. 0.8.2 [77] to draw fitted values from the posterior distribution of the HGAM.

### (d) Phenology trends

To assess trends in flight activity, we used a linear mixed model with species and vegetation type as random slopes and intercepts. To determine whether species and vegetation type contributed significantly to phenological variation over time, we examined the variance components and performed a likelihood ratio test (LRT) comparing models with and without random slopes for species and vegetation type.

Similarly, we examined whether shifts in muscid fly activity correlated with air temperature and snowmelt timing. To account for within-subject variability, we mean-centred temperature for each species and vegetation type, and scaled both predictors by their standard deviation, enabling direct comparison of effect sizes [78]. Given the heterogeneity in sampling frequency, population numbers and the shape of phenology distribution curves, phenology estimates had variable credible intervals. To incorporate this uncertainty, estimates were weighted by the inverse of their standard errors.

While this approach does not estimate direct correlations between phenology and climate, we can still explore possible drivers of change. Taking this approach, we assume that the posterior distribution of the activity metrics derived are Gaussian distributed and, therefore, we can use the standard error of the mean as a good description of the relative error in each observation.

### (e) Species’ traits and trends in phenology

We assessed how phenological niche, body size and anthophilous behaviour influenced temporal trends in species’ flight season (electronic supplementary material, table S1.4 and figure S1.4). The mean date of peak activity throughout the study period served as the continuous metric for the phenological niche factor. We derived information on average wing length for *Spilogona* species from Michelsen [27], while for the remaining species: *D. groenlandica*, *D. segnis* and *P. bidentata*, data were extracted from images published on boldsystem.org. Anthophilous behaviour, based on flower visitation rates and plant species visited, was categorized as ‘frequent’ or ‘infrequent’ using data from Olesen *et al.* [79]. We also tested for a phylogenetic signal of phenological responses (see technical details in the electronic supplementary material, figure S1.8 and table S1.8).

## 3. Results

### (a) Model fit

The best-fit model, which allowed for the seasonal pattern of abundance to vary by year, species and vegetation type, demonstrated a good fit to the abundance data (electronic supplementary material, table S1.1 and figure S1.3). This model accounted for 87.9% of the deviance and exhibited high fidelity, while not forcing the model to a specific shape. The intergroup variability of species and vegetation type was best described by a four-level interaction in a tensor product smooth, as shown in the model above.

### (b) Phenology of the flight season

**Figure 1.**
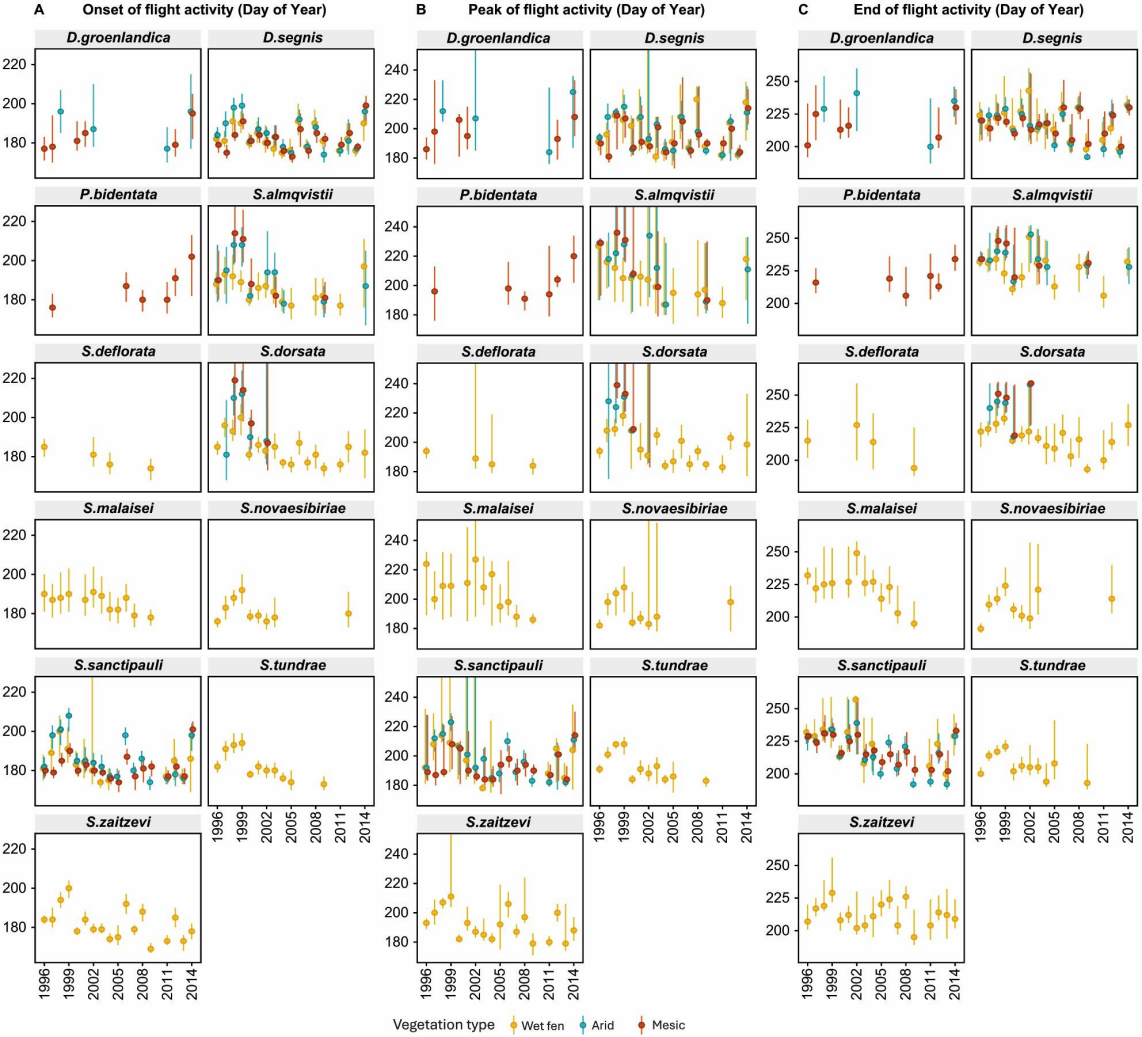
Temporal trends (1996–2014) in (A) onset, (B) peak and (C) end of the flight activity of muscid species across three vegetation types (wet fen, arid heath and mesic heath) at Zackenberg, NE Greenland. Error bars represent credibility intervals at the 95% level.

The muscid fly community flight season began in mid- to late June and lasted until the closing of the growing season in late August. Temporal variation in flight activity was evident across species and vegetation types. For instance, species like *S. zaitzevi* and *Spilogona novaesibiriae* (Frey 1915) displayed earlier annual flight activity, while *Spilogona almqvistii* (Holmgren 1880) and *Spilogona dorsata* (Zetterstedt, 1845) were active later in the season (electronic supplementary material, figure S1.4). Differences in activity patterns were also observed across vegetation types, with flies in the wet fen and arid heath displaying earlier annual flight activities than those in the mesic heath (figure 1).

On average, species demonstrated earlier flight activity over time (onset: −4.6 ± 0.9; peak: −8.5 ± 1.3; end: −7.1 ± 1.4 days/decade), with onset, peak and end of the season shifting up to several weeks per decade across species. The exception was *P. bidentata,* where the 6 years of observations available suggested delayed activity (figure 1). We found little evidence of interspecific variation in the onset of flight activity (species' responses did not deviate significantly from the average phenological response; LRT_onset_: *χ*² = 0.88, d.f. = 3, *p* = 0.83). However, species varied in the rate of phenological shift in the peak and end of their flight seasons (LRT_peak_: *χ*² = 14.94, d.f. = 3, *p* ≤ 0.01; LRT_end_: *χ*² = 28.90, d.f. = 3, *p* ≤0.001). For example, *S. almqvistii* shifted the peak of its flight season at greater rates than other species (−15.7 ± 1.4 days/decade). Notably, the trend towards earlier activity was most pronounced in the early years of the study, levelling-off in more recent years, suggesting a nonlinear trend in phenological shifts that is observed for many species in this fly community (figure 1). The two most abundant species, *D. segnis* and *S. sanctipauli*, showed substantial interannual variation in their onset of flight activity (onset*_D. segnis_*: *R*^2^ = 0.004; onset*_S. sanctipauli_*: *R*^2^ = 0.01), with rapid shifts towards earlier end of flight activity for *S. sanctipauli*, suggesting a shortened flight season. Across vegetation types, phenological responses were similar, with little variation in trends for the three most abundant species: *D. segnis*, *S. almqvistii* and *S. sanctipauli* (the random effect of vegetation type on the slope of phenology across years was <1% for all phenological events; electronic supplementary material, table S1.7).

### (c) Climatic impacts on phenology

Throughout the study period, Zackenberg experienced earlier snowmelt and rising air temperatures, particularly in the 30 days before the flight season ended (electronic supplementary material, table S1.8 and figure S1.6 & S1.7). Snowmelt timing, which varied substantially interannually, had a stronger influence on muscid fly phenology than temperature, consistently driving earlier flight activity (electronic supplementary material, figure S1.8). However, species responded at different rates (LRT_onset_: *χ*² = 46.10, d.f. = 6, *p* ≤ 0.001; LRT_peak_: *χ*² = 66.51, d.f. = 6, *p* ≤ 0.001; LRT_end_: *χ*² = 46.39, d.f. = 6, *p* ≤ 0.001), with *S. almqvistii*, *S. dorsata* and *S. malaisei* (Ringdahl 1920) among the most responsive. Temperature effects were weaker, but most pronounced at the end of the flight season, where higher temperatures in the 30 days prior were linked to an earlier end of flight (electronic supplementary material, figure S1.8). Some species also showed earlier flight onset in warmer years. While we did not explicitly assess flight season duration, our results suggest that higher temperatures may shorten the active period, potentially reducing fly longevity.

### (d) Phenology of species’ traits

**Figure 2.**
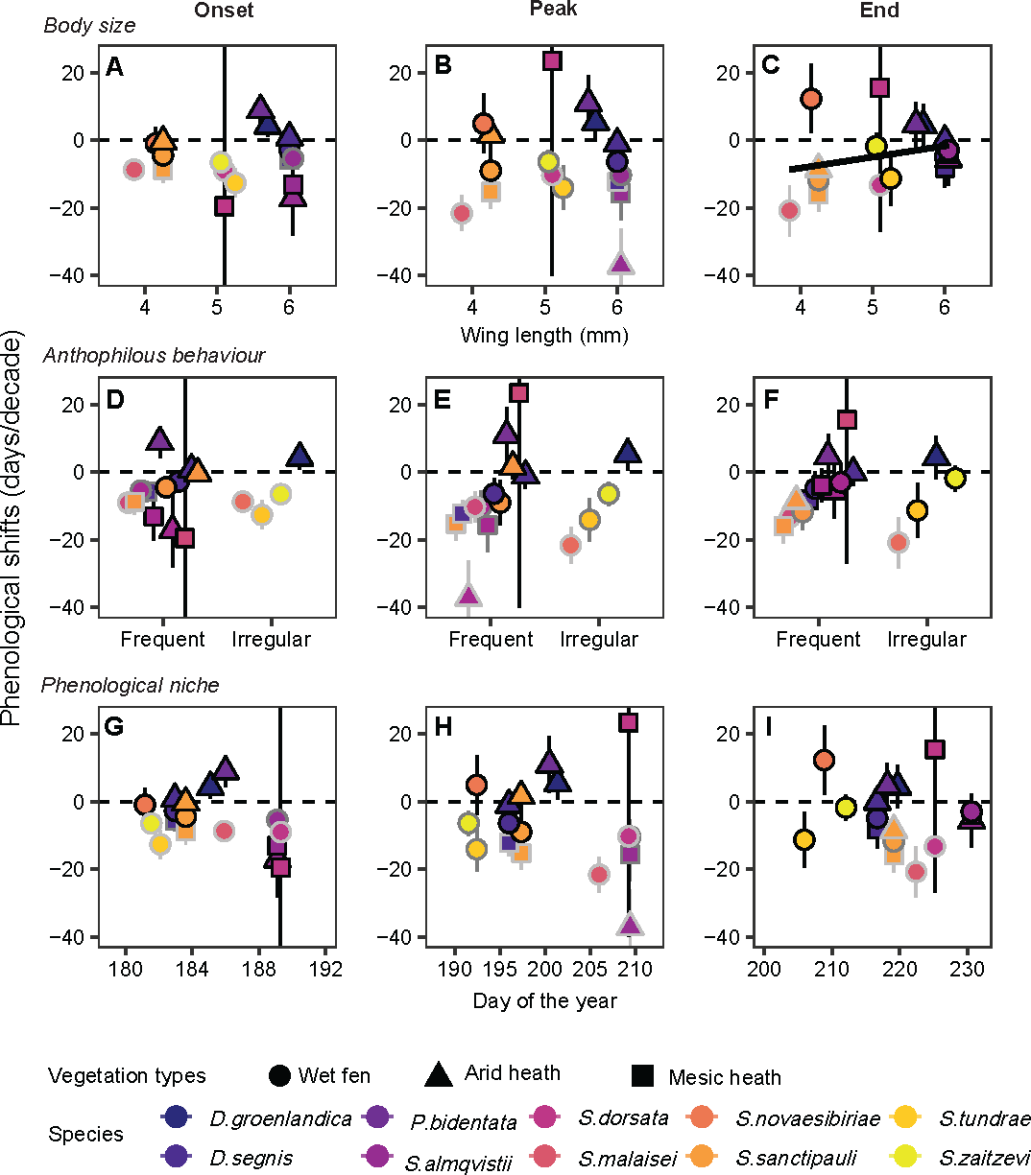
Temporal phenological shifts (days/decade) in onset, peak, end of flight activity for muscid fly species based on (A–B) body size (wing length, mm), (D–F) anthophilous behaviour and (G–I) phenological niche (average annual flight activity across all years). Positive values represent delayed phenology, negative values earlier phenology. Border colour indicates confidence level: light grey (95%), dark grey (90%) and black borders (non-significant). Species with <5 years of data (*Spilogona deflorata*, *D. groenlandica* in mesic heath, *S. dorsata* in arid heath) were excluded due to insufficient data. A significant positive trend in end of the flight season shifts with body size suggests smaller species advanced their flight season at greater rates than larger species over the 18 year study period.

Our analysis of species’ traits revealed that body size influenced temporal phenological shifts, with smaller flies advancing the end of their flight season at greater rates than larger flies. Notably, *S. malaisei* and *S. sanctipauli*, the two smallest species, exhibited the strongest advances (figure 2C). This suggests that smaller species gradually contract the duration of their flight activity across the study period. However, we found no evidence to suggest that species change their flight activity according to anthophilous habits or phenological niche (figure 2).

**Figure 3.**
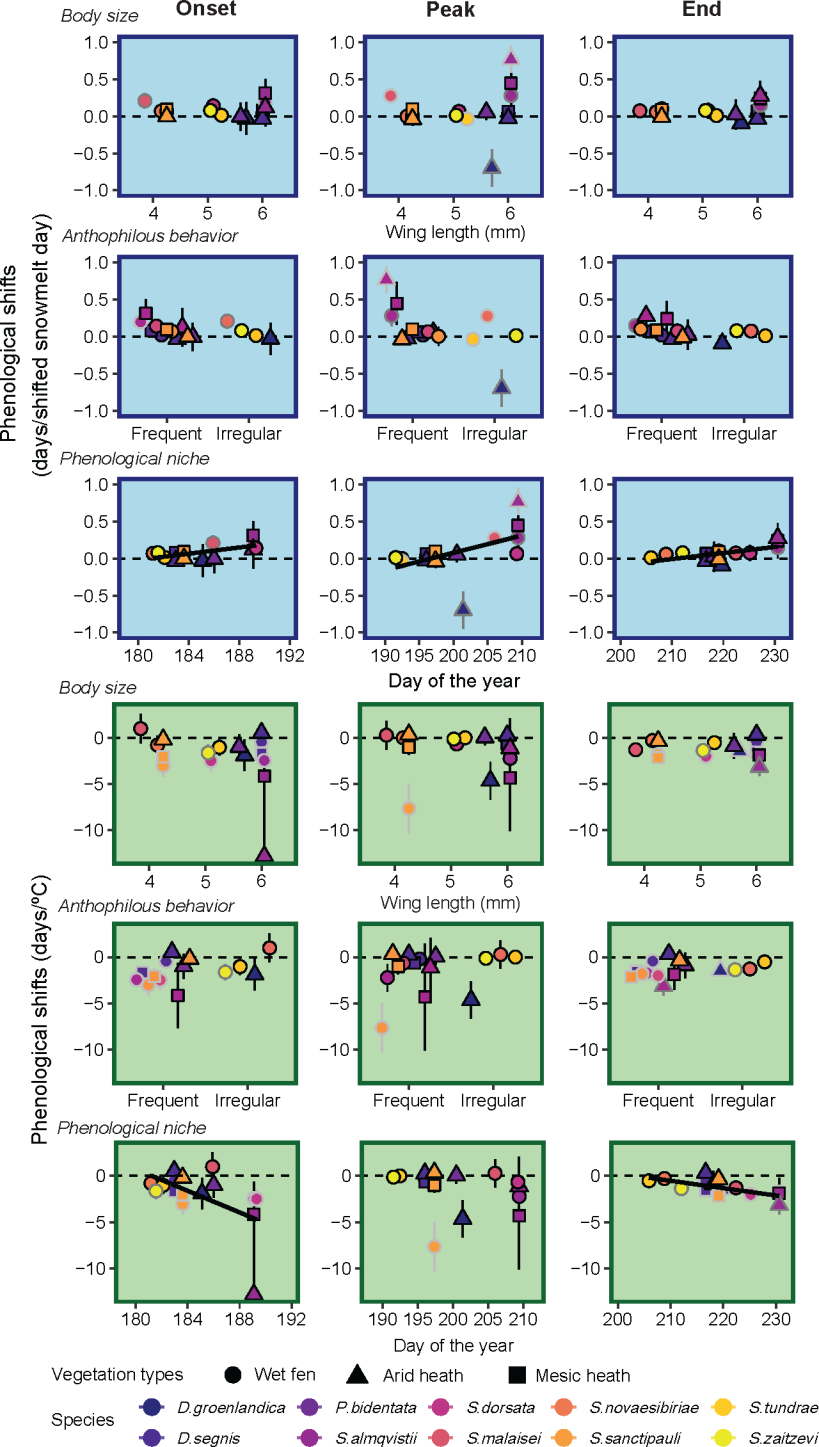
Phenological shifts in response to timing of snowmelt (blue panels) and air temperature 30 days prior to a mean phenological event minus 1 s.d. (green panels). Columns show onset, peak and end of flight activity across gradients of body size (wing length, mm), anthophilous behaviour and phenological niche (average annual flight activity across all years). Border colour indicates confidence level: light grey (95%), dark grey (90%) and black borders (non-significant). A strong trend was observed between phenological shifts and phenological niche (indicated by regression line).

In contrast, when examining species’ phenological niches, we found that the timing of annual flight activity influenced sensitivity to snowmelt timing and temperature (figure 3). Species with later annual flight activity (*S. almqvistii, S. dorsata* and *S. malaisei*) exhibited more pronounced shifts in both their onset and end of their flight activity in response to snowmelt timing and temperature than species with earlier flight activity (*D. segnis*, *S. novaesibiriae*, *S. tundrae* and *S. zaitzevi*). No clear trends in phenology–climate relations were found based on body size or anthophilous habits (figure 3). We did not find a significant phylogenetic signal in phenological responses, suggesting that responses are not predictable on the basis of relatedness among species (electronic supplementary material, figure S1.8 and table S1.8).

## 4. Discussion

Our analyses offer novel insights into the phenology and climate sensitivity of high-Arctic insects, revealing species-specific variations. By using a hierarchical modelling approach, we estimated phenological events throughout the adult muscid seasonal flight period, while accounting for intra-annual uncertainty and interannual phenological shifts. Most species exhibited a trend towards earlier flight activity over the 18-year study, likely driven by warmer temperatures and earlier snowmelt at Zackenberg. Interestingly, smaller flies advanced their end of the flight season more rapidly than larger ones, and late-active species exhibited stronger shifts in response to temperature and snowmelt timing than early-active species. These findings highlight differential sensitivities within the muscid fly community to environmental changes, with species' traits playing a critical role in shaping phenological responses. Such shifts could have implications for sustaining biotic interactions with ongoing climate change, especially plant–pollinator relationships, as muscids are key Arctic pollinators [20,54].

Our hierarchical modelling framework effectively captured detailed intraannual and interannual phenological patterns among species and vegetation types. The model’s flexibility enabled accurate predictions of flight activity across the phenological niche of each species–vegetation type combination available (figure 1), accounting for years with zero capture rates without imposing arbitrary shapes of abundance distributions. This provides robust, quantitative evidence of species-specific phenological dynamics, adding nuance to our understanding of how phenology varies within the muscid fly community under changing environments.

Between 1996 and 2014, the muscid fly community at Zackenberg shifted towards earlier flight activity, with onset, peak and end of flight activity advancing by up to several weeks. Smaller species, such as *S. malaisei* and *S. sanctipauli*, advanced the end of their flight activity more rapidly than larger ones, a pattern consistent with other studies linking body size to phenological shifts in response to temperature [3,80]. This could be attributed to smaller species having higher metabolic rates than larger ones [81], leading to faster development and earlier adult maturation, provided energy is efficiently allocated to development [82]. In addition, increased metabolism driven by warming could lead to reduced body size in insects [83], a trend observed for some species [84,85], which may have adverse effects on fitness [86] and dispersal capacity [87]. Given *S. sanctipauli*’s role as a pollinator in the Arctic [20], its strong phenological shifts could impact plant reproductive success if its flight timing outpaces plant flowering periods, increasing the risk of mismatch in Arctic ecosystems [19,88]. Such mismatches could be particularly problematic in Arctic ecosystems, where flowering seasons are short and alternative pollinators are limited [20].

Snowmelt timing was a key driver of phenology, likely through its effects on soil moisture [89,90] or by altering the amount of thermal radiation received [91,92]. Warmer temperatures following snowmelt can accelerate insect development rates [93], leading to earlier emergence. While no single temperature window was identified as the strongest predictor, flight season end dates were more sensitive to temperature than onset or peak, highlighting the importance of late-summer warming in phenological shifts. This ties with our finding that phenological sensitivity varied with species’ phenological niche. Late-season species, such as *S. almqvistii, S. dorsata* and *S. malaisei*, showed greater shifts in their onset and end of activity in response to climate than early-season species. This contrasts with findings from temperate regions [29,31,33], where late-season species often delay the end of their seasonal activity. This may be owing to faster warming in late summer compared with spring and early summer at Zackenberg over the 18-year period.

While our study primarily relies on broader climatic trends, the lack of detailed microhabitat data limits our ability to fully resolve how fine-scale environmental variation influences phenology. Microhabitat conditions, such as soil temperature, moisture levels and localized wind exposure, can create species-specific thermal niches that may mediate phenological shifts [94–96]. In Arctic environments, ground-level temperatures can vary significantly depending on snow cover persistence, vegetation structure and substrate type [97]. Without microclimate data, we may underestimate species’ ability to fine-tune their phenology to local thermal conditions. Future studies incorporating high-resolution microclimate measurements would improve our understanding of species-specific responses to climate change.

Our findings align with studies showing that warming shortens the activity periods of univoltine species, likely owing to temperature-driven reductions in adult lifespan (i.e. as temperature increases, lifespan decreases) [98–100]. A shorter activity period could have several demographic consequences, potentially reducing reproductive success by limiting time for mating and offspring production [101,102] and disrupting key ecological processes, such as pollination. For instance, these shifts may lead to a contraction of the community’s overall flight activity window as climate change progresses, potentially disrupting interactions between muscid flies and flowering plants. Despite differences in phenological sensitivity among muscid flies, flower-visiting species (e.g. *D. segnis*, *P. bidentata* and *S. sanctipauli*) did not respond differently from non-flower visitors, suggesting that species are not limited by the availability of flowering plants. Dominant plant species at Zackenberg have shifted their flowering periods more rapidly (onset: −9.3 ± 2.0; peak: −9.1 ± 1.2; end: −9.2 ± 1.6 days/decade; estimates from Iler *et al.* [103]) than muscid flies have shifted their flight periods (onset: −4.6 ± 0.9; peak: −8.5 ± 1.3; end: −7.1 ± 1.4 days/decade), yet overall synchrony appears maintained, likely owing to the generalist nature of both flies [20,48] and plants [79] in this high-Arctic community. This is further supported as the pollen transfer season has remained stable over the same 18-year period [19]. We therefore conclude that phenological mismatches probably have not occurred but this is a risk in the future if rates of warming increase.

Habitat can modulate phenological responses in insects, as different vegetation types are affected by climate change to varying degrees [45,46]. However, we did not find habitat-specific responses among muscid flies, despite evidence that wetlands—particularly the preferred habitat of many *Spilogona* species—are undergoing significant climate-driven changes [60,104]. Previous studies have documented muscid fly abundance declines in Zackenberg’s wet fen habitat [59]. The absence of habitat-specific responses in our study may be owing to most *Spilogona* species being found in the wet fen, while their numbers were too low in mesic and arid heath habitats to allow meaningful phenological comparisons. Nonetheless, localized habitat changes could still have significant consequences for their life cycle, as most muscid fly larvae rely on wet environments for development [27].

Family-level studies at Zackenberg suggest that phenological responses vary by vegetation type [11], but our species-level analysis suggests this may reflect differences in species' composition rather than direct habitat effects. Coarse taxonomic resolution can obscure whether phenological shifts stem from local climate conditions or distinct species assemblages. This suggests that studies relying on higher taxonomic levels may mask critical species-specific trends, a conclusion supported by research on butterfly and spider phenology and abundance at Zackenberg [10,105]. Muscid flies experience varying environmental conditions across life stages, complicating phenological analyses. Arctic species, typically those that are uni- or semivoltine (i.e. have generation times of at least 1 year) [106], terminate diapause, pupate and develop to the adult stage faster in warmer conditions [81,92,107], leading to earlier seasonal termination and potentially shorter adult longevity. In contrast, the commonly multivoltine insect species in temperate regions sometimes delay their season end to accommodate additional generations [108,109]. These findings underscore the complexity of insect phenological responses to climate change and emphasize the need for further research on entire life-cycle impacts [42,110].

While most muscid fly species experienced pronounced temporal shifts towards earlier flight activity, our dataset ends in 2014. Family-level arthropod studies at Zackenberg extending to 2023 show phenological responses levelling off [111], likely owing to increased interannual variability in snowmelt and slower early-summer warming. A similar pattern may apply to muscid species, but without more recent data, it remains unclear whether their phenological responses have continued or shifted direction over the past decade. Despite these uncertainties, our findings provide crucial insights into high-Arctic insect phenology over nearly two decades. We demonstrated substantial species-specific phenological variation in high-Arctic muscid flies over nearly two decades, linked to snowmelt timing and temperature. Traits such as body size and phenological niche emerged as key predictors of phenological responses, underscoring the importance of species-level analyses. However, limited taxonomic and ecological knowledge of Arctic muscids constrains our ability to interpret trait effects and detect ecological interactions. Addressing these gaps requires targeted natural history studies, expanded trait datasets and integration of species into ecological networks. Continued long-term monitoring and trait-based approaches are essential to better understand and predict the ecological consequences of climate-driven phenological change.

## Data Availability

All data used in this study are available from Dryad: [64]. This includes: — DNA sequences (originally downloaded from BOLD Systems: dx.doi.org/10.5883/DS-MOZK [accessed March 2025]) — Phylogenetic data — Phenology and climate data The R script to replicate all results and figures are available in a Zenodo repository: [112]. Supplementary material is available online [113].

## Authors' Notes

Throughout the study period, all animals were collected in accordance with annual permits and regulations from the government of Greenland under the non-exclusive license no. for utilization of Greenland genetic resources, granted to Arctic Research Center, Aarhus University (e.g., journal nos. G19−006, G21−005).
